# Primary and secondary dystonic syndromes: an update

**DOI:** 10.1097/WCO.0b013e3283633696

**Published:** 2013-07-03

**Authors:** Gavin Charlesworth, Kailash P. Bhatia

**Affiliations:** a Department of Molecular Neuroscience; b Sobell Department of Motor Neuroscience and Movement Disorders, UCL Institute of Neurology, London, UK

**Keywords:** cerebellum, dystonia, genetics, nonmotor, phenotype

## Abstract

**Purpose of review:**

The dystonias are a common but complex group of disorders that show considerable variation in cause and clinical presentation. The purpose of this review is to highlight the most important discoveries and insights from across the field over the period of the past 18 months.

**Recent findings:**

Five new genes for primary dystonia (*PRRT2*, *CIZ1*, *ANO3*, *TUBB4A* and *GNAL*) have made their appearance in the literature. New subtypes of neuronal brain iron accumulation have been delineated and linked to mutations in *C19orf12* and *WDR45*, while a new treatable form of dystonia with brain manganese deposition related to mutations in *SLC30A10* has been described. At the same time, the phenotypes of other forms of dystonic syndromes have been expanded or linked together. Finally, there has been increasing recognition of both the extramotor phenotype in dystonia and the part played by the cerebellum in its pathophysiology.

**Summary:**

Recently, there has been unprecedented change in the scientific landscape with respect to the cause of various dystonic syndromes that is likely to make a direct impact on clinical practice in the near future. Understanding the genetic cause of these syndromes and the often wide phenotypic variation in their presentations will improve diagnosis and treatment. With time, these discoveries may also lead to much-needed progress in elucidating the underlying pathophysiology of dystonia.

## INTRODUCTION

The dystonias are a heterogenous group of hyperkinetic movement disorders, characterized by involuntary sustained muscle contractions affecting one or more sites of the body that lead to twisting and repetitive movements or abnormal postures of the affected body part. They represent the third most common movement disorder worldwide [1–4]. There are currently 23 DYT loci referring to primary dystonic syndromes, as well as a number of primary dystonic syndromes not currently assigned to any DYT loci (see Table 1). In addition, there are numerous heredodegenerative disorders in the context of which dystonia is commonly seen.

Recent advances in the field of dystonia have mirrored to a considerable degree advances in genetic technology. Fuelled by the increasing use of whole-exome sequencing approaches, researchers have been able to elucidate the genetic cause of various familial forms of dystonia at an unprecedented rate. In doing so, several new dystonic syndromes have come into existence, whereas other long-recognized dystonic syndromes have been linked either to novel or occasionally even well-known genes. These discoveries are addressed in the following sections.

## PAROXYSMAL KINESIGENIC DYSKINESIA: PRRT2 AND PHENOTYPIC HETEROGENEITY

Paroxysmal kinesigenic dyskinesia (PKD), which is also known as DYT10, is a form of primary dystonia with a prevalence of about one in 150 000 individuals [5]. It is characterized by frequent (up to 100 times a day) attacks of dystonic or choreiform movements lasting from a few seconds to a few minutes. In late 2011, mutations in *PRRT2* (proline-rich transmembrane protein 2) were identified as the cause of PKD in nearly all cases [6,7]. Most mutations are truncating and by far the most common of these is the c.649dupC mutation, but missense variants (possibly with reduced penetrance) have also been described [8–11]. Interestingly, the c.649dupC mutation was also found in the family used to define DYT19, a supposedly separate form of PKD, suggesting that the initial linkage in this family was incorrect and DYT19 is in fact synonymous with DYT10 [12].

**Box 1:**
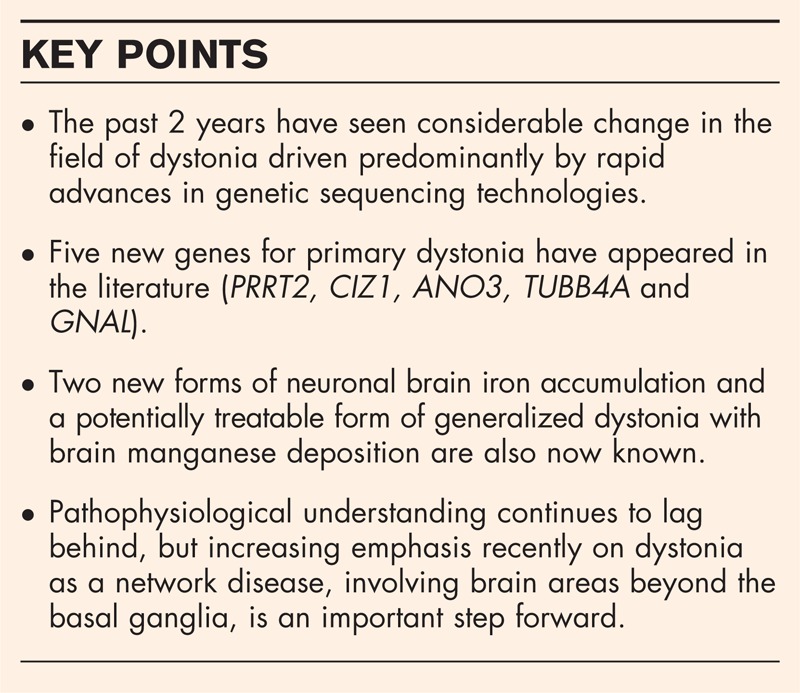
no caption available

The gene encodes a protein which is highly expressed throughout the nervous system. On a cellular level, it appears that the PRRT2 protein is predominantly localized to the synapse and that it may participate in neurotransmitter release, suggesting disrupted neurotransmission as the possible pathological mechanism in PKD [13].

Most interesting of all, perhaps, has been the recognition that mutations in this gene also cause a number of other paroxysmal disorders. In fact, *PRRT2-*related disease appears to be a stunning example of the kind of phenotypic heterogeneity often encountered in genetic forms of dystonia, with clinical presentation differing not merely between individuals carrying different mutations, but also between individuals carrying the same mutation and even between affected members within the same family [14]. Two forms of childhood epilepsy, namely, infantile convulsions with choreoathetosis (ICCA) and benign familial infantile seizures, as well as episodic ataxia, hemiplegic migraine and benign paroxysmal torticollis of infancy are all now recognized as possible manifestations of mutations in this gene [12,15–17].

## EXPANDING PHENOTYPES IN DYSTONIA

In a similar fashion to *PRRT2*, the phenotype of mutations in *ATP1A3* has recently been revised to include what was previously considered a separate condition. Mutations in this gene are traditionally associated with rapid onset dystonia-parkinsonism. In the last few months, however, it has become clear that *ATP1A3* mutations are also responsible for a neuropaediatric condition labelled alternating hemiplegia of childhood (AHC) [18^▪^,19^▪^]. AHC is a rare disorder characterized by the onset before the age of 18 months of paroxysmal neurological events, such as hemiplegia alternating in laterality, quadriplegia, dystonic spells, oculomotor abnormalities and autonomic dysfunction, all of which abate during sleep [20]. Nonparoxysmal manifestations develop after a few months or years of the disease, comprising developmental delay, intellectual disability of variable degree, ataxia, dysarthria, choreoathetosis, and, in some patients, pyramidal tract signs. Two separate studies were able to demonstrate de-novo heterozygous mutations in *ATP1A3* as the cause of the disorder in 75–100% of their case cohorts [18^▪^,19^▪^].

Meanwhile, the phenotypic spectrum of mutations in *SGCE*, which causes myoclonus dystonia, has also been revised to reflect an increased awareness of psychiatric manifestations of the disease. One recent study systematically assessed 27 *SGCE* mutation carriers using a battery of standardized psychiatric interviews and questionnaires and compared them with a disability-matched control group of patients with alcohol-responsive tremor [21^▪^]. Obsessive–compulsive disorder was eight times more likely and generalized anxiety disorder and alcohol dependence five times more likely in *SGCE* mutation carriers than in the tremor controls. This well designed study indicates that *SGCE* mutations are associated with a specific psychiatric phenotype consisting of compulsivity, anxiety and alcohol misuse, beyond the typical motor manifestations [21^▪^].

Finally, primary dystonia itself has undergone a phenotypic expansion of sorts in light of growing evidence indicating an important nonmotor component to the condition, including abnormalities in sensory and perceptual functions as well as neuropsychiatric, cognitive and sleep domains. These additional features may help explain the fact that reduction in quality of life in dystonia does not always correlate with the extent of the motor deficit. Moreover, it may be possible to capitalize on some of these extramotor signs – impaired temporal discrimination thresholds, for instance – to detect nonmanifesting mutation carriers in familial forms of dystonia, which would greatly facilitate the identification of new genes wherein establishing segregation is key. Interested readers are directed to a recent review by Stamelou *et al.*[22^▪^] and the paper by Kimmich *et al.*[23].

## FOUR NEW GENES FOR PRIMARY PURE DYSTONIA

In 2012, four new genes have been reported to cause primary pure dystonia, in which dystonia is the sole manifestation with the exception of tremor (see Table 2). Most studies used to identify the new genes have employed a whole-exome sequencing approach combined with linkage analysis to tackle smaller kindreds than was previously possible.

The first gene to appear on the scene was *CIZ1*. A missense mutation in this gene was published as the likely cause of focal, adult-onset cervical dystonia, variably associated with mild tremor, which was inherited as an autosomal dominant trait with reduced penetrance [24^▪^]. The age of onset in the index family was quite wide, ranging from 18 to 66 years, but generalization was not observed. Screening in a cohort of patients with adult-onset dystonia identified two additional missense mutations in three individuals, all with focal cervical dystonia developing in mid-to-late life [24^▪^]. However, segregation analysis was not possible in any of these additional cases, somewhat compromising the level of genetic evidence for this gene. In addition, others have raised questions regarding the quality and coverage of the exome data, meaning it will be particularly important to await independent confirmation of mutations in this gene before it can be fully accepted as a definite cause of dystonia [25]. The protein encoded by *CIZ1* is a p21^Cip1/Waf1^-interacting zinc finger protein that is expressed in the brain and involved in DNA synthesis and cell-cycle control [26].

Mutations in a second gene, *ANO3*, were identified as the cause of autosomal dominant craniocervical dystonia in a moderately sized white kindred [27^▪▪^]. Subsequent screening of the whole gene in a cohort of 188 individuals with cervical dystonia revealed five further novel of variants, of which segregation could be demonstrated for two. Clinically, patients with mutations in this gene exhibited focal or segmental dystonia, variably affecting the craniocervical, laryngeal or brachial regions. There was often dystonic tremor with a jerky quality affecting the head, voice or upper limbs. The age at onset ranged from the very early childhood to 40 years of age. As with *CIZ1*, the dystonia was never seen to generalize in any affected individual, remaining confined to the head, neck and upper limbs. Indeed, some individuals with mutations in this gene manifested upper limb tremor alone and had been misclassified as essential tremor [27^▪▪^]. Little is known about the function of *ANO3*, but it is most highly expressed in the striatum and is thought to encode a Ca^2+^-activated chloride channel [27^▪▪^,^28^]. Such channels are believed to have a role to play in modulating neuronal excitability, suggesting a possible mechanism by which altered channel function could lead to aberrant neuronal excitability manifest as dystonic movements [29].

*TUBB4A* has recently been identified by two groups independently as the cause of DYT4 dystonia, also known as whispering dysphonia [30^▪^,31^▪^]. The condition was first described by forensic psychiatrist Neville Parker [32] in a large family with third decade onset of autosomal dominantly inherited laryngeal dystonia with generalization. Over 25 affected individuals have since been reported, with some exhibiting a distinctive ‘hobby horse’ gait [33]. Alcohol responsiveness is not uncommon, leading to severe alcohol abuse in some DYT4 patients [33]. To date, however, no other family with this characteristic phenotype has been described, suggesting that the condition is very rare. *TUBB4A* encodes β-tubulin-4a, a constituent of microtubules, and the mutation in this family (p.Arg2Gly) results in an arginine to glycine amino-acid substitution in the key, highly conserved autoregulatory MREI (methionine–arginine–glutamic acid–isoleucine) domain of the protein. One further novel variant in this gene (p.Ala271Thr) was detected in an individual exhibiting spasmodic dysphonia with oromandibular dystonia and dyskinesia with an age at onset of 60 [30^▪^]. However, segregation analysis was not possible, meaning the pathogenicity of the variant is uncertain.

Finally, mutations in *GNAL* have recently been identified as a cause of autosomal dominant primary dystonia and subsequently confirmed by a second group [34^▪▪^,35^▪^]. Initially, it appeared that *GNAL* may have been an exceptionally common cause of familial dystonia with the first group reporting that six out of only 39 families screened (∼15%) haboured mutations in this gene [34^▪▪^]. However, the second group found only three mutations in 760 individuals screened (<0.5%), which is perhaps more in line with what might be expected [35^▪^]. Regardless, clinical characterization of individuals carrying mutations in *GNAL* showed a strong cervical predilection, with 82% having onset in the region of the neck and 93% displaying cervical involvement by the time they were examined for the study in question. However, progression to other sites had occurred in at least half of those affected and generalized dystonia was seen in about 10% of cases [34^▪▪^]. Thus, the clinical phenotype in *GNAL*-related dystonia appears to be not dissimilar to that caused by mutations in *THAP1*. The gene is highly expressed in the olfactory epithelium and hyposmia was noted in some affected individuals, though not consistently [35^▪^]. *GNAL* encodes Gαolf, the alpha subunit of triheteromeric G protein Golf, which is involved in dopamine (D1) signalling [36]. As D1 receptors have a known role in mediating locomotor activity, the link between *GNAL* and dystonia is biologically plausible.

## A NEW WILSON'S DISEASE?

Recently, the first inborn error of manganese metabolism has been identified, clinically resembling Wilson's disease. Biallelic mutations in the gene *SLC30A10*, which encodes a manganese transporter, were shown to be the cause of a syndrome that consisted of early-onset generalized dystonia, liver cirrhosis, polycythemia, and hypermanganesaemia [37^▪▪^,^38^^▪▪^]. Serum manganese levels are generally in the range 1000–6000 nmol/l (normal <320) and MRI of the brain typically shows hyperintensities in the basal ganglia as well as the subthalamic and dentate nuclei. Over 20 affected individuals from 10 different families have been described by two separate groups.

As with Wilson's disease, the disorder appears at least partially treatable. Using a combination of ethylenediaminetetraacetic acid and ferrous fumarate, clinicians were able to reduce serum manganese and induce improvement in dystonia in patients with this condition [39]. Again, as with Wilson's disease, early introduction of treatment is likely to be key so that evaluation of serum manganese may also take its place alongside copper and caeruloplasmin studies as one of the few obligatory investigations in young-onset dystonia of unexplained cause.

## NEW FORMS OF NEURODEGENERATION WITH BRAIN IRON ACCUMULATION

Neurodegeneration with brain iron accumulation (NBIA) results from excessive iron deposition in the brain, mainly the basal ganglia. Pantothenate kinase-associated neurodegeneration (PKAN, NBIA1) and *PLA2G6*-associated neurodegeneration (PLAN, NBIA2) are the core syndromes, but several other less frequent genetic causes have been identified (including *FA2H*, *ATP13A2*, *CP* and *FTL*) [40]. Now, two further subtypes have been identified and linked to mutations in the genes *C19orf12* and *WDR45*.

Mutations in *C19orf12*, an orphan mitochondrial protein, appear to be a relatively common cause of NBIA, accounting for 23 of 161 idiopathic cases (∼15%) screened by one group [41]. The condition, dubbed mitochondrial membrane protein-associated neurodegeneration or MPAN, is characterized by cognitive decline progressing to dementia, prominent neuropsychiatric abnormalities and a motor neuronopathy [42]. Dystonia is common (∼75%) and frequently affects the hands and feet, although it is generalized in some. On MRI, the ’eye-of-the-tiger’ sign is absent, but iron deposition is seen in the globus pallidus and the substantia nigra. As with PLAN, cortical Lewy bodies were present on neuropathological examination of brain tissue from an affected individual [42].

*WDR45* is located on the X chromosome and encodes a beta-propeller scaffold protein with a putative role in autophagy. In late 2012, mutations in this gene were linked to a novel form of NBIA, now known by the acronym SENDA (static encephalopathy of childhood with neurodegeneration in adulthood). SENDA is characterized by global developmental delay in early childhood and slow motor and cognitive gains until adolescence or early adulthood, at which point dystonia, parkinsonism, and dementia supervene [43^▪^,44^▪^]. A unique feature of this form of NBIA was reported to be T1 hyperintensity surrounding a central linear region of signal hypointensity within the substantia nigra and cerebral peduncles. From a genetic point of view, the condition is interesting for at least two reasons. First, all mutations occurred *de novo* such that Mendelian inheritance was never observed. Second, although *WDR45* is located on the X chromosome and undergoes inactivation, the clinical features of this form of NBIA do not follow a pattern typical of an X-linked disorder. Instead, the phenotype of affected males is indistinguishable from that of females. It is thought that the most likely explanation for this is that males with germline mutations are nonviable and that postzygotic mutations are instead responsible for the condition in males [44^▪^].

## DISTINGUISHING PSYCHOGENIC FROM NONPSYCHOGENIC DYSTONIA

Accurately distinguishing psychogenic from nonpsychogenic, primary dystonia is essential if patients are to receive the form of treatment that is most likely to benefit them and progress is to be made with regard to understanding the underlying neurobiology. Two recent studies have sought reliable markers to distinguish these conditions. The first investigated cortical plasticity using transcranial magnetic stimulation and the paired associative stimulation protocol in patients with psychogenic or organic dystonia and compared them with healthy controls [45]. Whereas cortical plasticity was increased in the group with organic dystonia (as expected), it was normal in both patients with psychogenic dystonia and healthy controls. A second study used functional neuroimaging to investigate movement-related cerebral activation in three groups, one with psychogenic fixed dystonia, another with genetically determined organic dystonia and a third consisting of healthy controls [46^▪^]. In organic dystonia, averaged regional cerebral blood flow was increased in the primary motor, premotor and parietal cortices, whereas it was decreased in the cerebellum. In contrast, patients with psychogenic dystonia displayed an opposite pattern of activation, with increased regional blood flow in the cerebellum and basal ganglia and decreased flow in the primary motor cortex. Together these studies suggest that distinct neurobiological mechanisms underpin organic and psychogenic dystonia, with the former related to aberrant motor cortical plasticity and metabolism and the latter related to abnormalities of frontosubcortical processing [46^▪^].

## THE RISING IMPORTANCE OF THE CEREBELLUM IN DYSTONIA

In terms of our understanding of the pathophysiology of dystonia, researchers have continued to make slow but steady progress. One notable trend in the last year has been a growing recognition of the part played by the cerebellum in the disorder. Multiple convergent strands of evidence from animal models, clinical observations, and anatomical, imaging and electrophysiological studies have highlighted the importance of the cerebellum in dystonia. At present, it remains unclear whether the cerebellar abnormalities observed in some of these studies are secondary (and perhaps compensatory) to basal ganglia dysfunction or are, in some cases at least, the driving force behind the development of dystonia. A recent review by Sadnicka *et al.*[47^▪^] of the accumulating evidence in this area as well as its implications is recommended to the interested reader.

## CONCLUSION

Fuelled by the rapid improvement in genetic sequencing technologies, there has been an unprecedented advance in our understanding of the cause of several forms of primary and heredodegenerative dystonia. It is likely that the continued improvement of these technologies – in particular the introduction of inexpensive whole-genome sequencing – will lead to further advances in coming years. At present, many of the new genes identified have yet to be independently confirmed and their prevalence as a cause of dystonia is as yet unclear. Nonetheless, an understanding of the function of these new genes may help researchers identify key pathways in the development of dystonia that could form the targets of novel therapeutic agents or interventions. Indeed, there remains an urgent need to clarify the underlying pathophysiology of the disorder, which is still poorly understood despite considerable research and effort. This is likely to be a difficult task given the apparent heterogeneity of causes in dystonia, but it is probable that a shift away from the paradigm of dystonia as a disorder of the basal ganglia alone towards one of dystonia as the common manifestation of dysfunction at any one of a number of points in a complex network of brain areas will prove an important step in this process.

## Acknowledgements

K.P.B. holds grants from the Bachmann-Strauss Dystonia and Parkinson Foundation, the Dystonia Society UK and the Halley Stewart Trust. This work was funded by a grant from the Bachmann-Strauss Dystonia and Parkinson Foundation.

### Conflicts of interest

K.P.B. has received honoraria/financial support to speak/attend meetings from GSK, Boehringer-Ingelheim, Ipsen, Merz, and Orion pharmaceutical companies. G.C. has no conflicts of interest to disclose.

## REFERENCES AND RECOMMENDED READING

Papers of particular interest, published within the annual period of review, have been highlighted as:

▪ of special interest
▪▪ of outstanding interest

Additional references related to this topic can also be found in the Current World Literature section in this issue (pp. 453–454).

## Figures and Tables

**Table 1:** The current DYT loci (after removal of withdrawn, duplicate and unpublished loci) with associated phenotype, mode of inheritance and genetic cause or linkage interval, wherever known

Locus symbol	Gene or linkage interval (wherever known)	Phenotype	Mode of inheritance
DYT1	*TOR1A*	Early-onset primary torsion dystonia with high prevalence in Jewish populations	AD
DYT2	Not known	Early-onset primary dystonia with prominent craniocervical involvement and generalization	AR
DYT3	*TAF1*	Adult-onset dystonia-parkinsonism, prevalent in the Philippines	X-linked
DYT4	*TUBB4A*	Whispering dysphonia with generalization, ’hobby horse’ gait and alcohol sensitivity	AD
DYT5a	*GCH1*	Progressive dopa-responsive dystonia with diurnal variation often affecting the lower limbs	AD
DYT5b	*TH*	Akinetic rigid syndrome with dopa-responsive dystonia or complex encephalopathy	AR
DYT6	*THAP1*	Adult-onset torsion dystonia with prominent craniocervical, laryngeal involvement and generalization	AD
DYT7	18p	Adult-onset primary cervical dystonia with questionable linkage	AD
DYT8	*MR-1*	Paroxysmal nonkinesigenic dyskinesia with attacks induced by alcohol, chocolate and stress	AD
DYT10	*PRRT2*	Paroxysmal kinesigenic dyskinesia with wide phenotypic variability, including epilepsy, migraine and intermittent torticollis	AD
DYT11	*SGCE*	Myoclonic dystonia, often with alcohol responsiveness, and psychiatric manifestations	AD
DYT12	*ATP1A3*	Rapid-onset dystonia parkinsonism or alternating hemiplegia of childhood	AD/*de novo*
DYT13	1p36.32–p36.13	Early-onset torsion dystonia in one Italian family only	AD
DYT15	18p11	Myoclonic dystonia with alcohol responsiveness in one Canadian kindred only	AD
DYT16	*PRKRA*	Early-onset dystonia-parkinsonism with a rostocaudal gradient	AR
DYT17	20p11.2–q13.12	Primary focal dystonia with progression in one Lebanese family only	AR
DYT18	*SLC2A1*	Paroxysmal exercise-induced dyskinesia with or without other features, such as epilepsy, haemolytic anaemia or spastic paraparesis	AD
DYT20	2q31	Paroxysmal nonkinesiogenic dyskinesia 2, in one large Canadian family only	AD
DYT21	2q14.3–q21.3	Adult-onset mixed dystonia with generalization in one Swedish family only	AD
DYT23	*ANO3*	Autosomal dominant, often tremulous craniocervical dystonia +/− upper limb tremor	AD

**Table 2:** Summary of the four new forms of primary pure dystonia identified in the last year

Gene name	Implicated mechanism	Typical age at onset by life stage	Typical distribution at onset	Tendency to generalize	Distinctive clinical features
*CIZ1*	Cell cycle regulation and DNA replication	Adult	Cervical	No generalization	Only reported in pure focal cervical dystonia
*ANO3* (DYT23)	Ca^2+^-activated chloride channel; striatal neuronal excitability?	Adolescence to early adulthood	Craniocervical or brachial	No generalization	Prominent head, voice or arm tremor
*TUBB4A (*DYT4)	Microtubule formation	Adolescence to early adulthood	Laryngeal, craniocervical	Frequent generalization	Ataxic, hobby horse gait; extrusional tongue dystonia; observed in a single family only
*GNAL*	Dopamine transmission via D1 receptors	Adolescence to mid-life	Craniocervical	Generalization in about 10%	Hyposmia?
